# Supplementary material for: Adverse Crosstalk between Extracellular Matrix Remodeling and Ferroptosis in Basal Breast Cancer
Source: Cells. 2023 Aug 30;12(17):2176. doi: 10.3390/cells12172176 (PMC10486747; doi:10.3390/cells12172176)
Supplement: Supplementary file 1 [file cells-12-02176-s001.zip › cells-2466896-supplementary.pdf]

Adverse Crosstalk Between Extracellular Matrix Remodeling and Ferroptosis in Basal Breast Cancer

Christophe Desterke [1,*], Emma Cosialls [2,3], Yao Xiang [2], Rima Elhage [2,3], Clémence Duruel [2,3], Yunhua Chang [2] and Ahmed Hamaï [2,3,*]

Supplemental material

Supplemental Figures

Figure S1: Kaplan-Meier of combined adverse 170 ferroptosis-related genes; Figure S2: Differential expression analysis between normal control breast and primary/metastatic breast cancer in RNAseq; Figure S3: Multiroc analysis of the eleven-gene signature against the TNBC phenotype; Figure S4: Multiroc analysis of the eleven-gene signature against dld-30 and residual cancer burden; Figure S5: Kaplan-Meier of the optimal age category stratification; Figure S6:Schoenfeld test of the multivariable model with DRFS as the outcome ;

Supplemental Table

Table S1: List of univariate Cox analysis for the 252 ferroptosis-related significant genes against Distant Relapse-Free Survival in the transcriptome cohort (GSE25066)

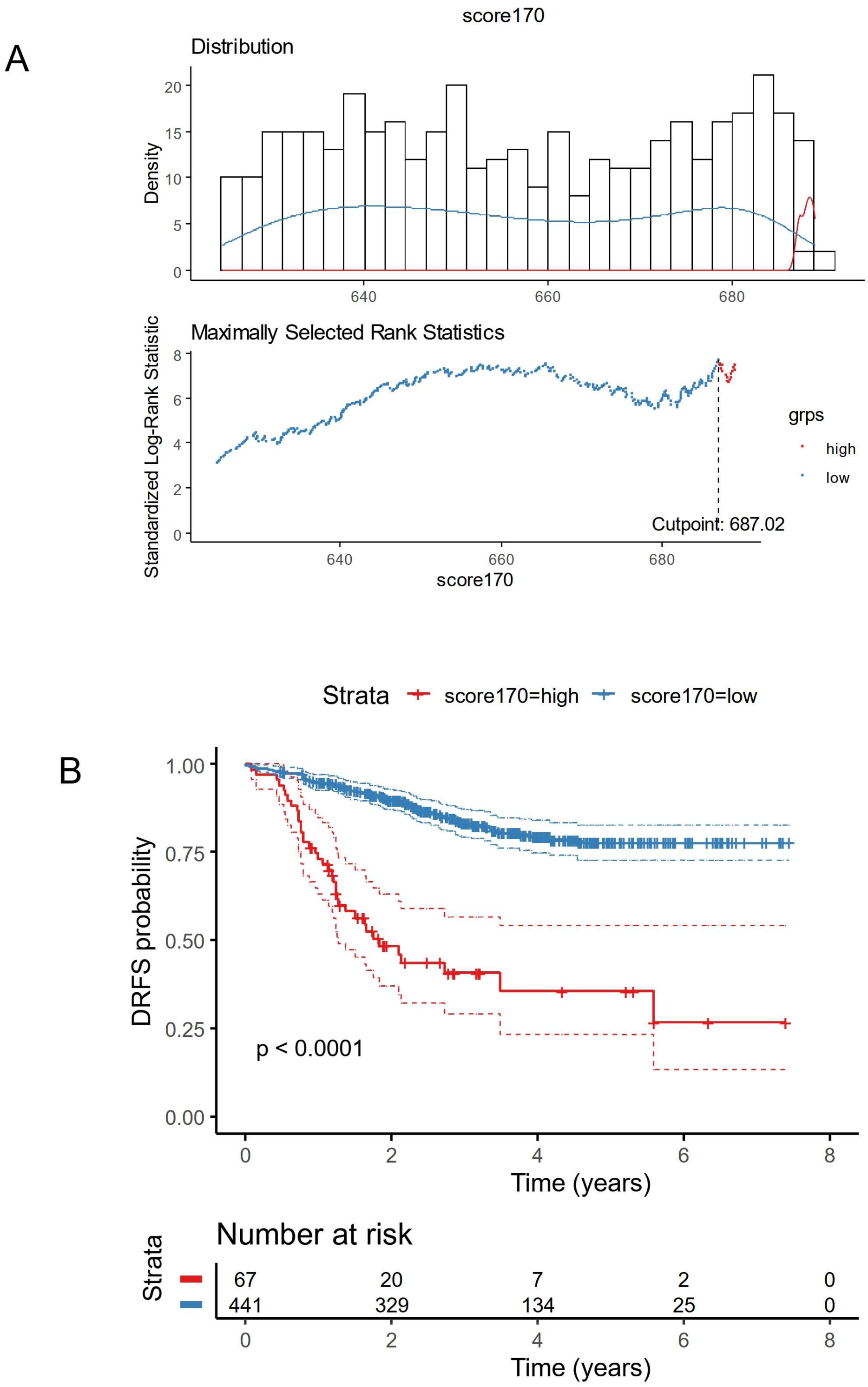

Figure S1: Kaplan-Meier of combined adverse 170 ferroptosis-related genes: A/ optimal cutpoint threshold determination on 170 ferroptosis adverse gene score with distant relapse-free survival (DRFS) as the outcome; B/ Kaplan-Meier stratified on optimal threshold of 170 ferroptosis adverse gene score.

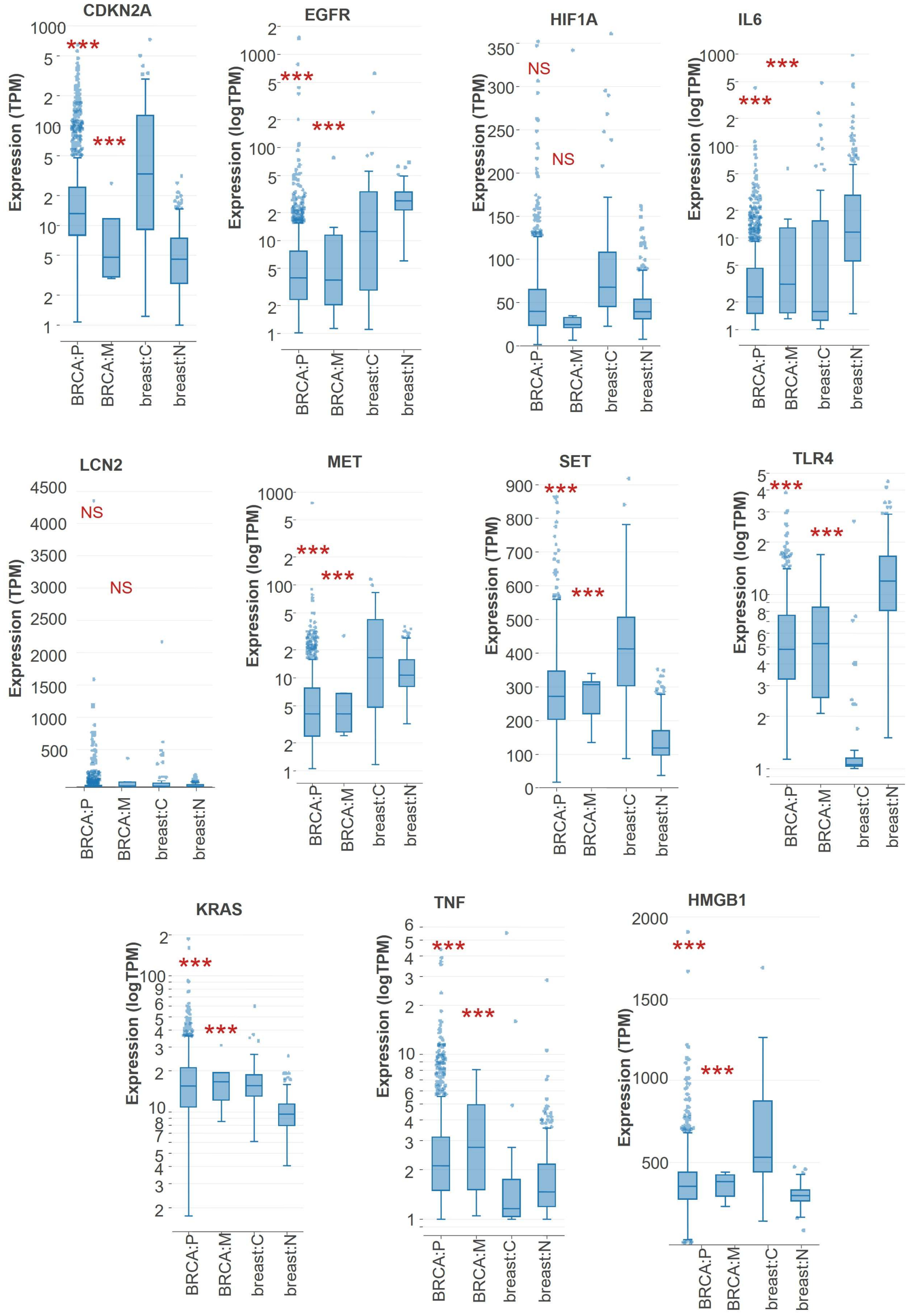

Supplemental Figure 2: Differential expression analysis between normal control breast and primary/metastatic breast cancer in RNAseq: For each eleven genes selected in score of ferroptsis/ECM remodeling in breast is expression in primary (BRCA:P) / metastatic (BRCA:M) breast cancer was compared to control normal breast (breast:N) by aggregation of TCGA/GTEX transcriptome on MiPanda server (remark: breast:C corresponds to breast cell lines from CCLE). significance: *: 0.01<p<0.05, **: 0.001<p<0.01, ***: p<0.001.

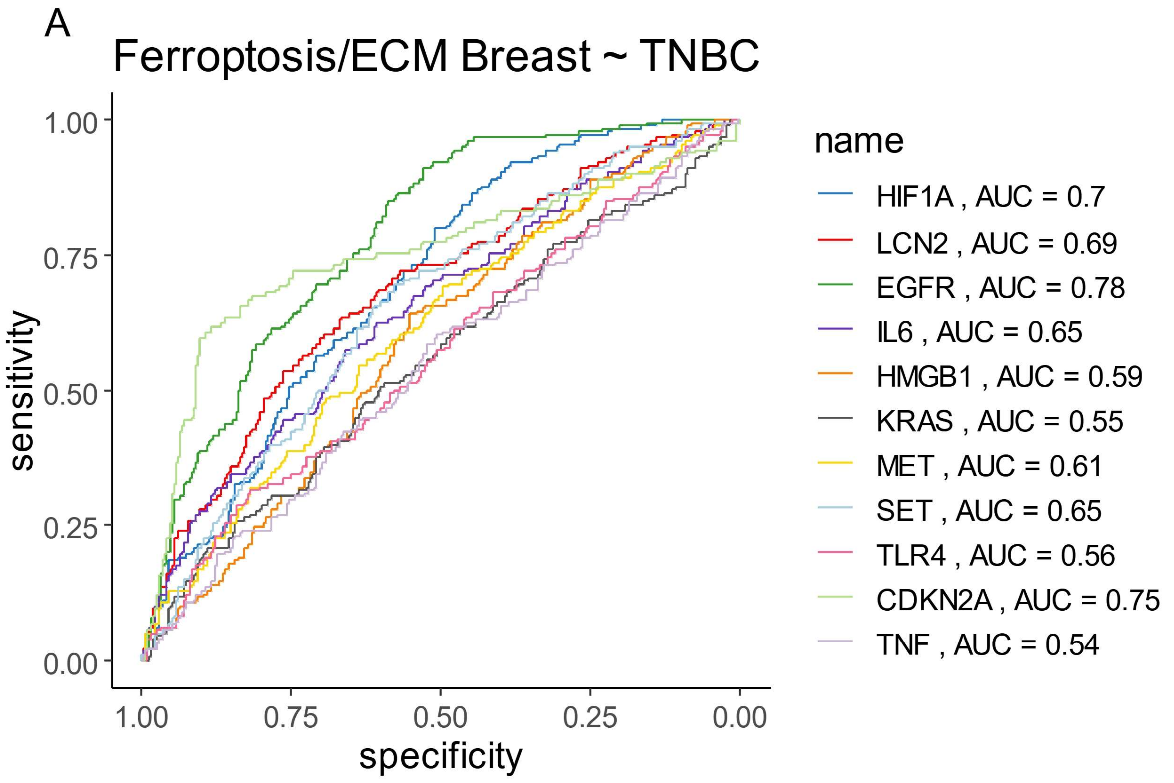

Supplemental Figure 3: Multiroc analysis of the eleven-gene signature against the TNBC phenotype

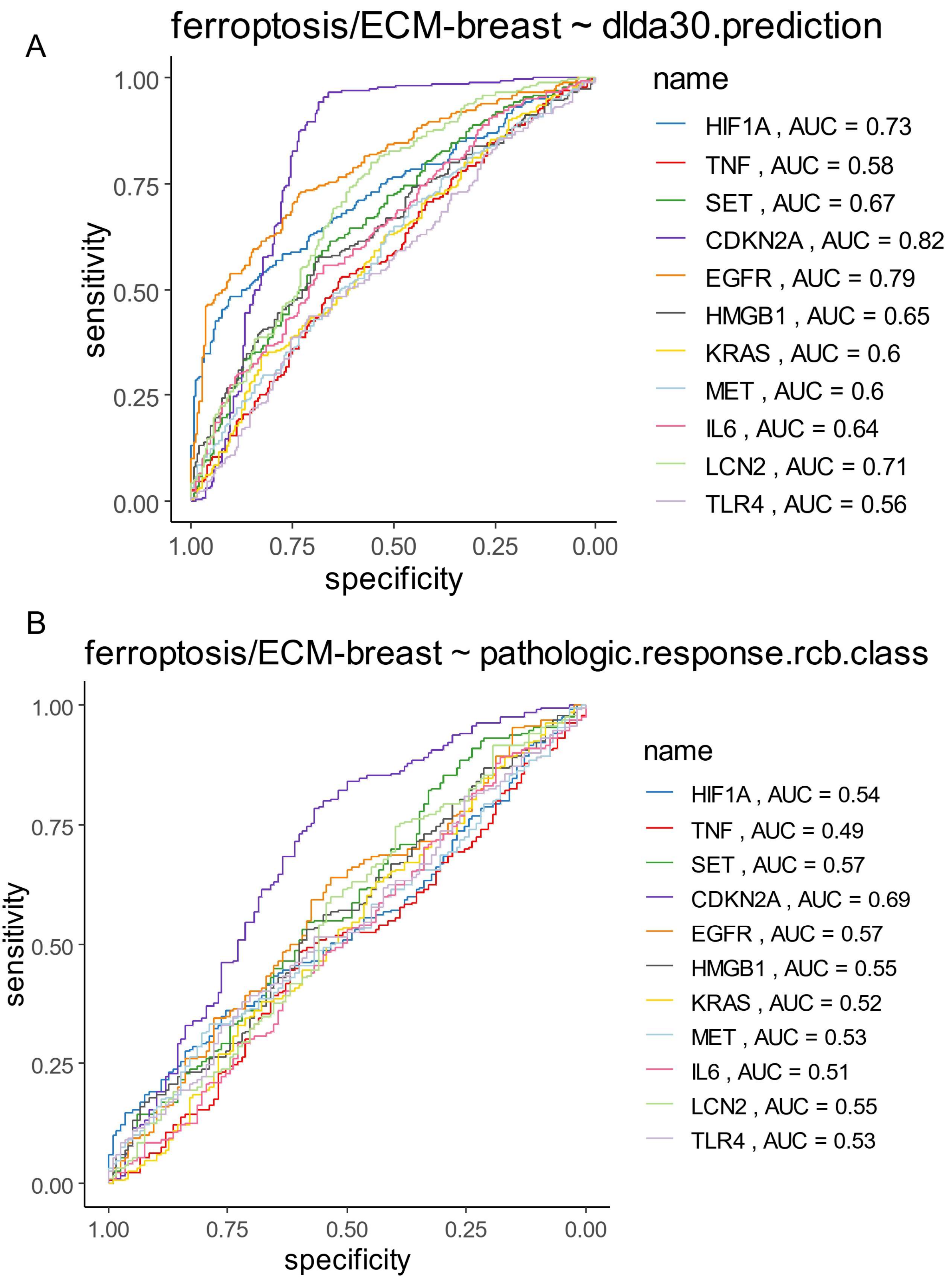

Supplemental Figure 4: Multiroc analysis of the eleven-gene signature against dld-30 and residual cancer burden

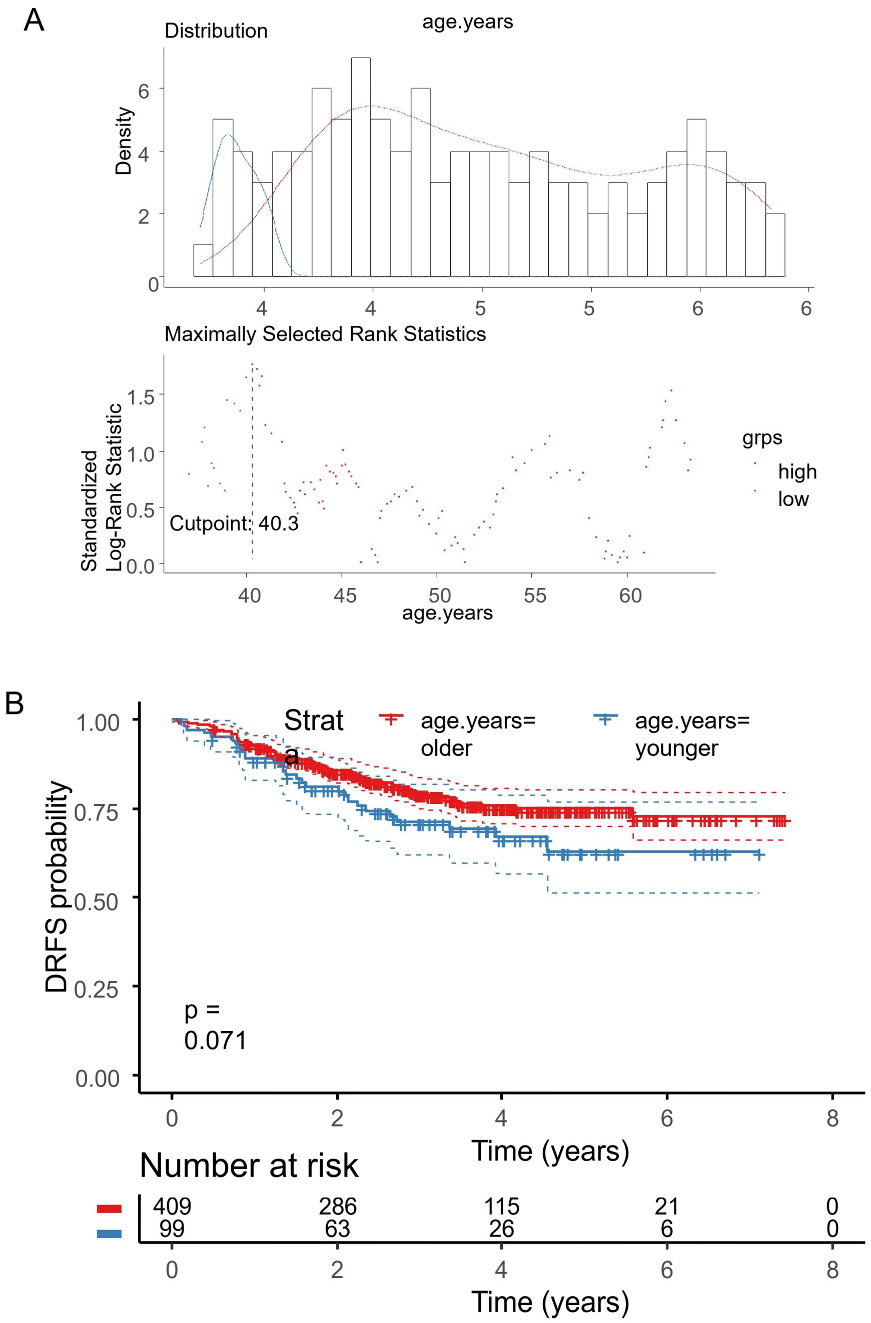

Supplemental Figure 5: Kaplan-Meier of the optimal age category stratification; A/optimal cutpoint threshold determination on age of the patients with distant relapse free survival (DRFS) as outcome; B/ Kaplan-Meier stratified on optimal threshold of age categories.

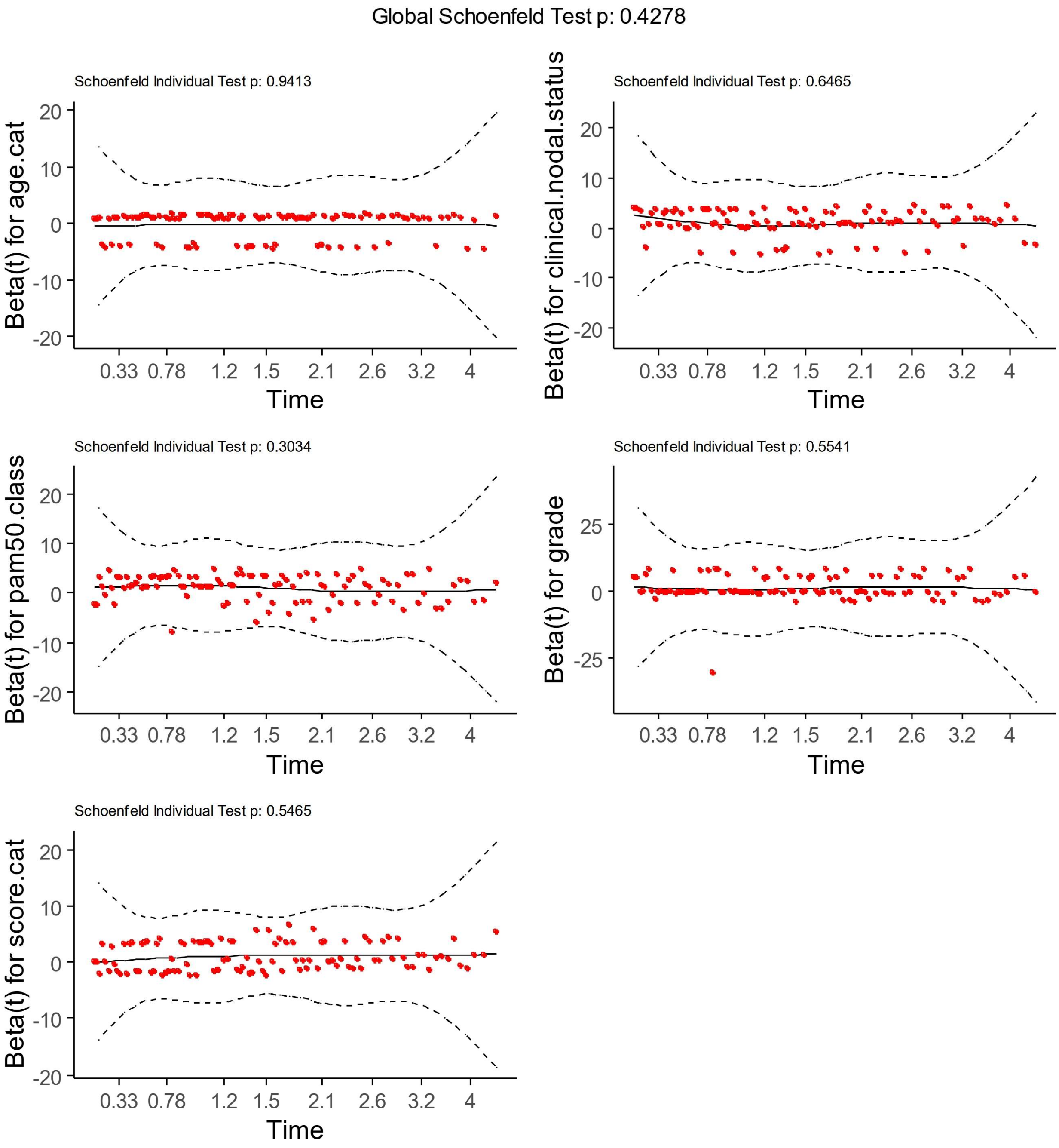

Supplemental Figure S6: Schoenfeld test of the multivariable model with DRFS as the outcome

Table S1: List of the univariate Cox analysis for the 252 ferroptosis related significant genes against Distant Relapse-Free Survival in the transcriptome cohort (GSE25066)

identifiers	Coefficients beta	Hazard Ratios	P-values	Adjusted p-vals
AR	-0,381	0,683	7,55E-12	6,52E-09
PTTG1	0,792	2,207	1,45E-09	4,99E-07
GATA3	-0,284	0,753	1,73E-09	4,99E-07
MYB	-0,392	0,676	2,97E-09	6,41E-07
XBP1	-0,381	0,683	1,12E-08	1,93E-06
ENO1	0,802	2,230	2,06E-08	2,63E-06
SLC7A5	0,411	1,508	2,13E-08	2,63E-06
AGR2	-0,135	0,874	2,66E-08	2,88E-06
LRIG1	-0,414	0,661	3,39E-08	3,25E-06
IRS1	-0,329	0,719	4,69E-08	4,05E-06
ITCH	1,348	3,849	5,50E-08	4,32E-06
ESR1	-0,236	0,790	8,54E-08	5,71E-06
SLC9A3R1	-0,519	0,595	8,59E-08	5,71E-06
EGLN1	0,813	2,255	1,18E-07	7,30E-06
RHOB	-0,456	0,634	2,00E-07	1,15E-05
BTG2	-0,635	0,530	2,63E-07	1,42E-05
SIAH2	-0,281	0,755	3,93E-07	1,94E-05
EGFR	0,363	1,438	4,04E-07	1,94E-05
INPP4B	-0,299	0,741	6,08E-07	2,77E-05
MST1	-0,442	0,643	8,12E-07	3,51E-05
FOXA1	-0,234	0,791	9,21E-07	3,79E-05
MYD88	0,810	2,247	1,17E-06	4,57E-05
FGFR3	-0,228	0,796	1,22E-06	4,57E-05
ERBB3	-0,494	0,610	1,64E-06	5,89E-05
TGFA	0,547	1,727	1,70E-06	5,89E-05
CEBPB	0,528	1,696	1,95E-06	6,48E-05
YBX1	0,533	1,704	2,81E-06	9,00E-05
ODC1	0,450	1,568	3,46E-06	1,05E-04
DDIT3	0,926	2,524	3,53E-06	1,05E-04
CCNG2	-0,510	0,601	3,90E-06	1,12E-04
NDRG1	0,315	1,371	4,48E-06	1,25E-04
DUSP4	-0,185	0,831	4,81E-06	1,25E-04
SDCBP	0,511	1,667	4,82E-06	1,25E-04
LCN2	0,243	1,275	4,92E-06	1,25E-04
TTK	0,357	1,429	5,32E-06	1,26E-04
CYCS	0,731	2,078	5,44E-06	1,26E-04
MCAM	0,519	1,681	5,52E-06	1,26E-04
BCL2	-0,381	0,683	5,53E-06	1,26E-04
PLAUR	0,461	1,585	5,87E-06	1,28E-04
CXCL8	0,194	1,215	5,91E-06	1,28E-04
LYN	0,376	1,456	6,07E-06	1,28E-04
IGF1R	-0,338	0,713	6,70E-06	1,38E-04
ERBB4	-0,361	0,697	6,87E-06	1,38E-04
MELK	0,360	1,433	9,74E-06	1,91E-04
SPDEF	-0,262	0,769	1,08E-05	2,07E-04
ADM	0,359	1,432	1,12E-05	2,10E-04
RARA	-0,303	0,739	1,15E-05	2,11E-04
CDC20	0,329	1,390	1,38E-05	2,49E-04

VEGFA	0,319	1,375	1,68E-05	2,96E-04
AREG	-0,201	0,818	1,73E-05	2,98E-04
ANO1	-0,222	0,801	1,82E-05	3,09E-04
HMGA1	0,378	1,459	2,00E-05	3,32E-04
KIF14	0,475	1,608	2,07E-05	3,38E-04
PROM1	0,196	1,216	2,64E-05	4,17E-04
AURKA	0,466	1,593	2,65E-05	4,17E-04
CDK1	0,443	1,557	2,89E-05	4,46E-04
CALR	0,712	2,039	3,92E-05	5,94E-04
PRLR	-0,389	0,678	4,18E-05	6,23E-04
CCNE1	0,326	1,385	4,61E-05	6,75E-04
RRM2	0,416	1,515	4,80E-05	6,90E-04
MET	0,685	1,983	4,91E-05	6,90E-04
DAPK1	0,492	1,636	4,95E-05	6,90E-04
CXCL1	0,291	1,338	5,18E-05	7,10E-04
VAV3	-0,310	0,734	5,26E-05	7,10E-04
ATF4	0,843	2,323	5,62E-05	7,47E-04
MAPK3	-0,287	0,751	7,38E-05	9,58E-04
ADAM17	0,658	1,931	7,52E-05	9,58E-04
PIM1	0,409	1,505	7,54E-05	9,58E-04
SLC9A1	-0,262	0,770	7,73E-05	9,68E-04
TFRC	0,379	1,462	8,11E-05	9,90E-04
IL1B	0,347	1,415	8,13E-05	9,90E-04
LDHA	0,728	2,070	9,67E-05	1,16E-03
PGR	-0,183	0,833	9,93E-05	1,17E-03
HMMR	0,665	1,944	9,99E-05	1,17E-03
SMURF1	0,694	2,003	1,05E-04	1,21E-03
PRMT5	-0,265	0,767	1,08E-04	1,23E-03
RUNX3	0,495	1,640	1,45E-04	1,62E-03
MKI67	0,694	2,001	1,49E-04	1,64E-03
GPNMB	0,278	1,321	1,52E-04	1,64E-03
IGF2BP3	0,278	1,321	1,52E-04	1,64E-03
LAPTM4B	0,347	1,415	1,59E-04	1,70E-03
NEDD4	-0,391	0,676	1,71E-04	1,79E-03
FOXC1	0,268	1,308	1,74E-04	1,79E-03
CDH3	0,158	1,171	1,74E-04	1,79E-03
DEK	0,371	1,449	1,94E-04	1,97E-03
TPX2	0,395	1,484	1,99E-04	1,99E-03
SPHK2	-0,320	0,726	2,10E-04	2,08E-03
LOXL2	0,291	1,337	2,12E-04	2,08E-03
AKT3	0,395	1,485	2,16E-04	2,09E-03
E2F3	0,555	1,743	2,25E-04	2,16E-03
ANXA1	0,263	1,301	2,34E-04	2,22E-03
FSCN1	0,252	1,287	2,38E-04	2,24E-03
ITGB1	0,532	1,702	2,43E-04	2,26E-03
PLAU	0,339	1,404	2,47E-04	2,27E-03
PRDX3	-0,524	0,592	2,60E-04	2,36E-03
CD9	-0,295	0,745	2,81E-04	2,53E-03
HDGF	0,550	1,733	2,97E-04	2,63E-03
UBE2C	0,382	1,465	2,98E-04	2,63E-03
DDIT4	0,306	1,359	3,12E-04	2,69E-03
SREBF1	-0,341	0,711	3,12E-04	2,69E-03

TXNRD1	0,493	1,637	3,17E-04	2,71E-03
IKBKB	-0,393	0,675	3,24E-04	2,75E-03
GPX4	-0,617	0,540	3,31E-04	2,78E-03
HTATIP2	0,400	1,492	3,40E-04	2,82E-03
STUB1	-0,656	0,519	3,56E-04	2,93E-03
KRT18	-0,260	0,771	3,78E-04	3,08E-03
UCHL1	0,221	1,248	3,86E-04	3,12E-03
ALCAM	-0,248	0,780	4,28E-04	3,42E-03
RHOC	-0,487	0,614	4,34E-04	3,44E-03
CHEK1	0,443	1,557	4,88E-04	3,82E-03
EIF4EBP1	0,279	1,321	4,98E-04	3,82E-03
SLC2A1	0,453	1,574	5,01E-04	3,82E-03
CCND1	-0,195	0,823	5,02E-04	3,82E-03
YWHAZ	0,551	1,735	5,04E-04	3,82E-03
ITGA3	-0,200	0,819	5,35E-04	4,02E-03
NEDD8	-0,574	0,563	5,63E-04	4,19E-03
PRKDC	0,486	1,625	5,77E-04	4,26E-03
WWP1	-0,295	0,744	5,96E-04	4,36E-03
FYN	0,341	1,406	6,02E-04	4,37E-03
SEMA4D	0,348	1,416	6,16E-04	4,43E-03
PLK1	0,367	1,444	6,22E-04	4,44E-03
HDAC2	0,363	1,438	6,81E-04	4,76E-03
BECN1	-0,538	0,584	6,82E-04	4,76E-03
MMP7	0,160	1,174	6,84E-04	4,76E-03
GAPDH	0,636	1,888	6,88E-04	4,76E-03
HIF1A	0,363	1,438	7,00E-04	4,80E-03
PML	0,622	1,863	7,09E-04	4,82E-03
CDKN2A	0,224	1,251	8,12E-04	5,48E-03
FASN	-0,271	0,763	8,25E-04	5,52E-03
TSC2	-0,236	0,789	8,49E-04	5,65E-03
GDF15	-0,174	0,840	8,75E-04	5,77E-03
SET	0,695	2,003	8,93E-04	5,84E-03
BTK	0,341	1,406	9,15E-04	5,94E-03
ATG5	0,446	1,562	1,04E-03	6,65E-03
SKP2	0,277	1,319	1,04E-03	6,65E-03
MMP14	0,450	1,568	1,09E-03	6,94E-03
CHI3L1	0,171	1,186	1,15E-03	7,21E-03
XAF1	0,303	1,354	1,15E-03	7,21E-03
TLR4	0,400	1,492	1,16E-03	7,21E-03
TRAP1	0,621	1,861	1,19E-03	7,35E-03
PYCARD	-0,258	0,772	1,25E-03	7,64E-03
AURKB	0,318	1,374	1,35E-03	8,23E-03
ESRRA	0,630	1,878	1,42E-03	8,56E-03
FOLR1	0,262	1,299	1,48E-03	8,89E-03
IGFBP2	-0,152	0,859	1,52E-03	9,04E-03
KCNH2	0,364	1,439	1,56E-03	9,21E-03
TFAP2A	-0,321	0,725	1,59E-03	9,29E-03
CXCR4	0,276	1,318	1,59E-03	9,29E-03
TNFRSF12A	0,409	1,505	1,61E-03	9,34E-03
CDKN3	0,456	1,578	1,65E-03	9,51E-03
ISG15	0,170	1,186	1,77E-03	1,01E-02
GSTP1	0,250	1,284	1,80E-03	1,02E-02

TRPM2	0,425	1,530	1,82E-03	1,03E-02
NEK2	0,251	1,285	2,08E-03	1,17E-02
TNF	0,372	1,450	2,12E-03	1,18E-02
CHEK2	0,448	1,565	2,15E-03	1,19E-02
TOP2A	0,265	1,303	2,17E-03	1,19E-02
TNFAIP3	0,320	1,377	2,22E-03	1,22E-02
YWHAQ	0,501	1,650	2,41E-03	1,31E-02
HES1	-0,333	0,717	2,54E-03	1,37E-02
SDC1	0,262	1,300	2,60E-03	1,39E-02
BMI1	-0,290	0,748	2,83E-03	1,51E-02
MAD2L1	0,254	1,290	2,87E-03	1,52E-02
IKBKG	0,527	1,695	2,90E-03	1,53E-02
HSPD1	0,483	1,621	2,92E-03	1,53E-02
XPO1	0,557	1,746	2,96E-03	1,54E-02
SLC16A1	0,271	1,311	3,11E-03	1,61E-02
YAP1	0,287	1,332	3,18E-03	1,63E-02
MSLN	0,095	1,100	3,27E-03	1,67E-02
ABCC4	0,222	1,249	3,32E-03	1,69E-02
MCL1	0,419	1,521	3,34E-03	1,69E-02
RPS6KB1	-0,295	0,744	3,37E-03	1,69E-02
ICAM1	0,362	1,436	3,48E-03	1,73E-02
CCNA2	0,331	1,392	3,49E-03	1,73E-02
TRIM28	-0,433	0,648	3,53E-03	1,74E-02
BIRC5	0,364	1,439	3,66E-03	1,80E-02
SPP1	0,179	1,196	3,73E-03	1,82E-02
NAMPT	0,223	1,250	3,75E-03	1,82E-02
SMYD3	-0,288	0,749	3,78E-03	1,82E-02
CFLAR	0,474	1,607	3,90E-03	1,87E-02
S100A7	0,075	1,078	3,98E-03	1,90E-02
MAP2K1	0,536	1,708	4,03E-03	1,91E-02
MAP3K14	0,370	1,448	4,05E-03	1,91E-02
TXN2	-0,578	0,561	4,18E-03	1,96E-02
PRKCA	0,301	1,351	4,20E-03	1,96E-02
PPP2CA	-0,478	0,620	4,32E-03	2,01E-02
DKK1	0,209	1,232	4,41E-03	2,03E-02
EPOR	0,349	1,417	4,42E-03	2,03E-02
HPSE	0,198	1,219	4,94E-03	2,26E-02
KRAS	0,546	1,726	5,01E-03	2,28E-02
MAP2K7	0,463	1,588	5,25E-03	2,36E-02
IL6	0,331	1,392	5,26E-03	2,36E-02
S100A9	0,126	1,135	5,26E-03	2,36E-02
TYMS	0,231	1,259	5,39E-03	2,40E-02
ETS2	0,334	1,396	5,52E-03	2,44E-02
PIK3R1	-0,276	0,759	5,67E-03	2,50E-02
ULK1	-0,221	0,802	5,74E-03	2,52E-02
MTA1	-0,458	0,633	5,83E-03	2,54E-02
E2F1	0,501	1,650	5,93E-03	2,57E-02
STAT6	-0,445	0,641	6,00E-03	2,59E-02
EZH2	0,214	1,239	6,30E-03	2,71E-02
IRF1	0,390	1,477	6,54E-03	2,80E-02
GJA1	-0,112	0,894	6,62E-03	2,80E-02
HBEGF	0,315	1,371	6,64E-03	2,80E-02

ADAM12	0,313	1,367	6,65E-03	2,80E-02
PEBP1	-0,420	0,657	6,81E-03	2,84E-02
LIMK1	-0,214	0,807	6,82E-03	2,84E-02
CCL5	0,201	1,223	6,83E-03	2,84E-02
PIK3CB	0,465	1,592	6,91E-03	2,85E-02
MAP3K5	0,366	1,442	7,01E-03	2,88E-02
NOTCH1	0,298	1,347	7,05E-03	2,89E-02
BAD	-0,204	0,816	7,10E-03	2,90E-02
CLU	-0,124	0,883	7,16E-03	2,91E-02
VIM	0,218	1,243	7,39E-03	2,98E-02
HMOX1	0,202	1,224	7,58E-03	3,05E-02
MARCKS	0,268	1,307	7,72E-03	3,09E-02
BIRC3	0,207	1,230	7,89E-03	3,14E-02
NLK	-0,370	0,691	7,92E-03	3,14E-02
CCNB1	0,314	1,369	8,06E-03	3,18E-02
KPNA2	0,292	1,340	8,09E-03	3,18E-02
MGMT	-0,335	0,715	8,25E-03	3,23E-02
CDC37	-0,188	0,829	8,33E-03	3,24E-02
MAPK9	-0,189	0,828	8,42E-03	3,26E-02
EDN1	0,161	1,175	8,60E-03	3,30E-02
ERCC1	-0,611	0,543	8,60E-03	3,30E-02
TKTL1	0,219	1,244	8,95E-03	3,41E-02
RAD51	0,291	1,337	8,99E-03	3,41E-02
NDUFA13	-0,377	0,686	9,00E-03	3,41E-02
FOXM1	0,171	1,186	9,08E-03	3,43E-02
BID	0,342	1,408	9,67E-03	3,62E-02
CD40	0,369	1,446	9,69E-03	3,62E-02
PRDX2	-0,352	0,703	9,79E-03	3,65E-02
HMGA2	0,322	1,379	1,00E-02	3,71E-02
PAWR	-0,328	0,720	1,00E-02	3,71E-02
VCAM1	0,198	1,219	1,01E-02	3,73E-02
RARB	0,352	1,421	1,07E-02	3,93E-02
S100A8	0,106	1,111	1,09E-02	3,99E-02
BIRC2	0,386	1,471	1,10E-02	4,00E-02
PRKCB	0,235	1,265	1,11E-02	4,00E-02
CCNA1	0,245	1,278	1,11E-02	4,00E-02
IGFBP3	0,152	1,164	1,14E-02	4,06E-02
PRKCD	-0,195	0,823	1,14E-02	4,06E-02
UBE2D1	0,297	1,346	1,15E-02	4,07E-02
COPS5	0,361	1,435	1,18E-02	4,19E-02
FOXO4	-0,182	0,834	1,20E-02	4,23E-02
THBS1	-0,197	0,821	1,25E-02	4,37E-02
CD82	0,276	1,318	1,25E-02	4,38E-02
VCAN	0,150	1,162	1,27E-02	4,41E-02
HMGB1	0,382	1,465	1,29E-02	4,49E-02
CDK8	0,334	1,396	1,32E-02	4,56E-02
SP1	-0,164	0,848	1,33E-02	4,58E-02
IDO1	0,176	1,192	1,42E-02	4,87E-02
